# The prevalence of primary ovarian insufficiency in Sweden; a national register study

**DOI:** 10.1186/s12905-018-0665-2

**Published:** 2018-10-25

**Authors:** Katarina Lagergren, Mats Hammar, Elizabeth Nedstrand, Marie Bladh, Gunilla Sydsjö

**Affiliations:** Division of Obstetrics and Gynecology, Department of Clinical and Experimental Medicine, Faculty of Medicine and Health Sciences, Linköping University, SE-58185 Linköping, Sweden

**Keywords:** Primary ovarian insufficiency, Register study, Prevalence

## Abstract

**Background:**

The current estimates of the prevalence of primary ovarian insufficiency (POI) are very variable, but are in most studies believed to be around 1%. It is also very likely tat the prevalence of POI differs between countries and over time. We therefore aimed to assess the prevalence of primary ovarian insufficiency in Sweden.

**Methods:**

All 1,036,918 women born between 1973 and 1993 in Sweden were included. The prevalence of POI was based on data from the Swedish Patient Register through the diagnosis code or through the Prescribed Drug Register. The number of women below 40 years of age diagnosed with the ICD-10 diagnoses E28.3 or E89.4, and women who had been dispensed drugs for treatment of climacteric symptoms were included.

**Results:**

Out of the 1,036,918 women, 19,253 (1.9%) had POI. The prevalence of spontaneous POI was 1.7% and the prevalence of iatrogenic POI was 0.2%. Most women (98.8%) with POI were identified from the Prescribed Drug Register; only 4.1% were found in the Patient Register, whereas 2.9% were identified in both registers.

**Conclusions:**

The total prevalence of POI was 1.9%, 95% CI: 1.7–2.1, indicating a higher prevalence than often previously reported.

## Background

Menopause is the permanent cessation of menstruation and is denoted “early menopause” if it occurs before the age of 45, and as primary ovarian insufficiency (POI) if occurring before the age of 40 [1, 2]. POI is classified as primary (spontaneous) or secondary (iatrogenic). The causes of spontaneous POI remain unknown in 90% of the cases, but may be caused by chromosomal abnormalities, gene mutations, autoimmunity disorders, metabolic disorders, and infections. The syndrome is a chronic disorder and may cause comorbidity such as depression and anxiety disorders because of the loss of reproductive hormones and the fact that these women face infertility the women may also suffer autoimmune adrenal insufficiency or hypothyroidism. Moreover, the risk of reduced bone mineral density and increased risk of cardiovascular disease related to estrogen deficiency is evident in the long term perspective for the women with POI [2].

Iatrogenic POI is typically caused by surgery, chemotherapy or radiotherapy, but the prevalence is very uncertain and probably differs between countries and over time. A previous study from the US found that spontaneous POI affected 1% (95% confidence interval 0.3–1.5%) of the female population under the age of 40 [3]. This study included a total of 1858 women born between 1928 and 1932, who were followed until they reached menopause. The type and date of menopause were recorded. In total, there were nine women with natural menopause before the age of 40, i.e. 1%. A register-based study conducted in Estonia [4] showed a prevalence of spontaneous POI of 0.91% (95% CI 0.81–1.02). However, a more recent pooled study, including 51,450 postmenopausal women from nine observational studies in the UK, Scandinavia, Australia, and Japan, showed a prevalence of premature menopause of 2% [5].

The objective of this study was to assess the prevalence of POI, spontaneous and iatrogenic, separately and combined in Sweden, where unselected and nationwide data on POI are available through well-maintained national registers.

## Methods

The data were collected from three nationwide Swedish health data registers, the Medical Birth Register for identification of women born 1973–1993, the Patient Register and the Prescribed Drug Register to identify women who had been diagnosed with POI. The personal identification number, a 10-digit number unique to each resident in Sweden, allowed linking information from the registers used on an individual level of all cohort members.

*The Medical Birth Register (MBR),* initiated in 1973, collects all information regarding pregnancies and childbirths.

*The National Patient Register (NPR)* records all inpatient care in Sweden since 1987, and all specialist outpatient care since 2001. This register includes data on diagnoses, external causes of injury, and surgical procedures. The diagnoses in the Patient Register are based on the Swedish version of the International Classification of Diseases (ICD). The eighth version (ICD-8) was used before 1986, ICD-9 between 1987 and 1996, and ICD-10 has been used since 1997. The Patient Register has been validated for its high completeness and accuracy regarding both diagnoses [6] and surgical procedures [7].

The *Swedish Prescribed Drug Register* includes information on all drugs prescribed and dispensed in Sweden since 1st July 2005. It contains information about all prescriptions, including doses and amounts.

This population-based cohort study of all women born in Sweden between 1973 and 1993 according to the MBR (*n* = 1,036,918) was designed to estimate the prevalence of POI, spontaneous and iatrogenic, separately and combined, during the period 1st July 2005 to 31st December 2013. Cases of POI were identified from two sources. The Patient Register was used to identify the ICD diagnosis codes defining POI: E28.3 and E89.4 (ICD-10). Individuals with POI were also identified from the Prescribed Drug Register by assessing prescriptions of systemic hormone replacement therapy (HRT) for climacteric symptoms among women younger than 40. This therapy included oral and transdermal products within the ATC groups G03CA03 oestradiol (excluding low-dose products and products for local vaginal treatment only), G03CA57 conjugated oestrogens, G03CX01 tibolone, G03FA01 norethisterone and oestrogen, G03FA12 medroxyprogesterone and oestrogen, G03FA15 dienogest and oestrogen, G03FA17 drospirenone and oestrogen, G03FB05 norethisterone and oestrogen, G03FB06 medroxyprogestgerone and oestrogen, and G03FB09 levonorgestrel and oestrogen. Since these drugs are only prescribed to treat menopausal symptoms and not for contraception, women prescribed these drugs and who were younger than 40 were considered to have POI. Since HRT therapy is also prescribed to patients with iatrogenic POI (e.g. after bilateral oophorectomy for cancer), all women with a cancer diagnosis were excluded when estimating the prevalence of spontaneous POI. These excluded women were identified in the Patient Register using the following ICD-codes: all diagnoses starting with C in ICD-10, codes 140–239 in ICD-9 and codes 140–239 in ICD-8. Women who had been dispensed HRT and were considered to have POI and who had undergone a bilateral oophorectomy (operation ICD-10 code LAF10) were also considered to have iatrogenic POI. To minimalize the risk of misclassification, we analysed how many dispensations the women had, and we conducted a separate analysis where only women with two or more dispensations were considered to have POI.

### Statistics

The total prevalence of POI, as well as the prevalence of POI within different registers, was presented with absolute numbers and percentages. A 95% confidence interval was calculated for the total prevalence of POI.

## Results

Out of the 1,036,918 women, 19,253 (1.9%; 95% CI: 1.7–2.1) had POI, of which 18,883 (98.1%) were identified in the Prescribed Drug Registry and 783 (4.1%) in the National Patient registry (Table 1). Of those with iatrogenic POI, three individuals were identified in all three registers, and no one with spontaneous POI was identified in more than one of the registers. Among all women with POI, 17,185 (89.3%) had spontaneous POI and 2068 (10.7%) had iatrogenic POI (Table 1), corresponding to a prevalence of 1.7% of spontaneous POI and 0.2% of iatrogenic POI.

**Table 1:** Number (%) of patients diagnosed with primary ovarian insufficiency (POI) in Sweden during 1973–1993, out of the total 1,036,918 women included in the study

POI	Inpatient care	Outpatient care	Patient Register^c^	Prescribed Drug Register	Total
	n (%)	n (%)	n (%)	n (%)	n (%)
Total	120 (0.60)	783 (4.10)	794 (4.10)	18,883 (98.10)	19,253 (100.00)
Spontaneous	4 (0.02)	116 (0.68)	120 (0.70)	17,117^a^ (99.60)	17,185 (100.00)
Iatrogenic	10 (0.50)	667 (32.20)	674 (32.60)	1,766^b^ (85.40)	2068 (100.00)

^a^drug usage without a cancer diagnosis

^b^drug usage with a cancer diagnosis

^c^Either inpatient care or outpatient specialist care

Out the total cohort of women with POI, 460 (2.4%) had undergone a bilateral oophorectomy. Of these, 325 were also identified in the Prescribed Drug Register as having been dispensed HRT, and three were identified in the NPR as having received the diagnosis of POI.

When excluding women with fewer than two dispensations, 9897 (0.9%; 95% CI 0.88–0.92) had POI. In addition, 3516 women (0.3%) had more than five dispensations, i.e. they must have received a new prescription according to the Swedish drug administration system (Table 2).

**Table 2:** Number (%) of dispensations of HRT to women below 40 years of age and therefore supposed to have a POI diagnosis

Number of dispensations	Number of individuals (%)
1	8986 (0.9)
2–4	6381 (0.6)
5-	3516 (0.3)
Total	18,883 (1.8)

## Discussion

In this national cohort study of 1,036,918 women born between 1973 and 1993 we found a total prevalence of POI of 1.9%. Of these women, 89.3% had spontaneous POI and 10.7% had iatrogenic POI based on the assumption that women diagnosed with POI and a cancer diagnosis or a diagnosis of bilateral salpingo-oophorectomy had iatrogenic POI. Also women who have undergone hysterectomy without oophorectomy may have disturbed ovulations and be diagnosed with POI but these cases may not be caught by the use of a register study since they are out patients. The vast majority of cases were identified from the Prescribed Drug Register using HRT rather than from the Patient Register.

The methodological advantages of the study include the nationwide and population-based design, the high validity and completeness of the databases, and the large size of the study cohort. A limitation is the risk of incomplete registration of POI in the NPR, since this register covers inpatient and outpatient specialized care but not the general health care, implying a risk of underestimation of the prevalence of POI. On the other hand, there is a risk of overestimation of the prevalence if some women have been prescribed HRT without having POI. However, since HRT is clearly not indicated for contraception, we consider this risk small. There is probably an under-diagnosis of POI using dispensed HRT as a measure, since some women with POI may have been treated with oral contraceptives instead of HRT, [8] and those are not identified in this study, except for those diagnosed in the Patient Register. Furthermore, there are probably women who do not seek medical care because of POI, and can therefore not be found in registers. It could be argued that women who had few dispensations of HRT might have had their prescription without an established true diagnosis of POI. However, this cannot be controlled for in a register study. Furthermore, we know from a previous Swedish study based on similar methods, that women with early menopause (before 45 years of age) use HRT for a very short period, on average for less than one year, and thus it is not surprising that many women have very few dispensations [9].

The cohort included 460 women with an SOE diagnosis who had undergone bilateral oophorectomy, which results in iatrogenic POI. Interestingly, only three of these women had received a POI diagnosis and only 42% used HRT. This suggests that there is great under-diagnosis and probably under-treatment among these women.

In the literature, it is said that the incidence of POI is 1%, based on only one clinical study. However, a more recent study showed an incidence of 2%, i.e. similar to the result of this study. That study was not registry-based. The researchers used questionnaires and asked the subjects when their menstruations stopped and why. It is difficult to say which study is more accurate. Overall, incidence studies are difficult to interpret and compare since they are from different countries and time frames and used different methods. In this registry-based study we probably missed many women due to the large underdiagnosis. However, there is also a risk of over-reporting since women may receive HRT for other causes – for instance in treatment of infertility also in cases with other diagnoses than POI.. In the retrospective studies the women had not been examined by a specialist and the cause of early menopause was not known. In conclusion, the present study shows a prevalence of 1.9%, indicating a higher prevalence of POI than previously often reported. However, substantial under-diagnosis remains likely.

The exact incidence of iatrogenic POI will therefore remain somewhat uncertain.

## Conclusions

In conclusion, POI is prevalent, apparently more prevalent than often previously reported. Since women with POI face serious health risks both mental and physical, they need thorough medical information, surveillance and hormonal treatment. POI also affects fertility, which with increasing age at first pregnancy may cause a need for assisted reproduction i.e. IVF and most possible need for oocyte donation.

## Abbreviations

ART: Assisted Reproductive Techniques
CI: Confidence Interval
HRT: Hormone Replacement Therapu
ICD: International Classification of Diseases
IVF: In-Vitro Fertilization
MBR: Medical Birth Register
NPR: National Patient Register
POI: Premature Ovarian Insufficiency
SOE: Salpingo-Oophorectomy
